# A Rare Case of Trigeminal Neuralgia Caused by an Aneurysmal Bone Cyst in the Temporal Bone

**DOI:** 10.7759/cureus.36846

**Published:** 2023-03-29

**Authors:** Ghadah J Khormi, Naif K Mahzara, Ammar W Baltoyour, Wed F Alhazmi, Ahlam Alharbi

**Affiliations:** 1 General Practice, Jazan University, Jazan, SAU; 2 General Practice, Imam Abdulrahman Bin Faisal University, Dammam, SAU; 3 General Practice, King Fahad Specialist Hospital, Tabuk, SAU; 4 Family Medicine, Primary Health Care Center, Riyadh, SAU

**Keywords:** case report, magnetic resonance imaging, aneurysmal bone cyst, petrous bone lesions, trigeminal neuralgia

## Abstract

Trigeminal neuralgia is a rare and debilitating condition characterized by severe facial pain, often caused by the compression or irritation of the trigeminal nerve. Although trigeminal neuralgia caused by petrous bone lesions is uncommon, it can significantly impact a patient's quality of life. In this case report, we describe a 40-year-old female with a five-year history of severe facial pain diagnosed as trigeminal neuralgia. Imaging revealed an aneurysmal bone cyst in the petrous part of the left temporal bone, located near the trigeminal nerve's root entry zone. The patient underwent a successful left retrosigmoid craniotomy with the resection of the lesion, resulting in a significant improvement in her symptoms. At the six-month follow-up, the patient reported no recurrence of her symptoms and a considerable improvement in her quality of life. Although trigeminal neuralgia caused by an aneurysmal bone cyst in the left temporal bone is rare, surgery is the most effective treatment. Long-term outcomes are generally favorable with close follow-up. This case report highlights the importance of early diagnosis and timely surgical intervention in the management of this debilitating condition.

## Introduction

Trigeminal neuralgia is a rare and debilitating condition characterized by recurrent episodes of severe facial pain, thought to be related to the compression or irritation of the trigeminal nerve at the root entry zone [1]. Aneurysmal bone cysts are rare benign bone tumors that can cause bony destruction and compression of adjacent neural structures. Although these tumors typically occur in individuals under the age of 30 and are more commonly found in the long bones, the spine, and the pelvis, aneurysmal bone cysts involving the skull base are rare [2]. Their clinical presentation can vary depending on the location and size of the lesion [2]. We present a case report of a patient who suffered from trigeminal neuralgia due to an aneurysmal bone cyst involving the petrous part of the left temporal bone. This case highlights the importance of considering structural causes in patients with refractory trigeminal neuralgia.

## Case presentation

Our patient was a 40-year-old female who had been experiencing severe facial pain for the past five years. The pain was described as a sharp, stabbing sensation that radiated from the left side of her face, including the forehead, cheek, jaw, and teeth. The pain was triggered by routine activities such as eating, brushing her teeth, or talking and could last for several seconds to a few minutes. She reported that the pain was so severe that it caused her to flinch, cry out, and avoid certain activities that could trigger the pain. The patient also reported that the pain had significantly impacted her quality of life.

The patient had sought medical attention for her symptoms and had been diagnosed with trigeminal neuralgia based on her clinical presentation and imaging studies. She had been treated with carbamazepine and gabapentin, but her symptoms persisted and worsened over time. On examination, the patient had no facial weakness, but there was evidence of decreased sensation to light touch and pinprick in the distribution of the left maxillary and mandibular branches of the trigeminal nerve.

**Figure 1:**
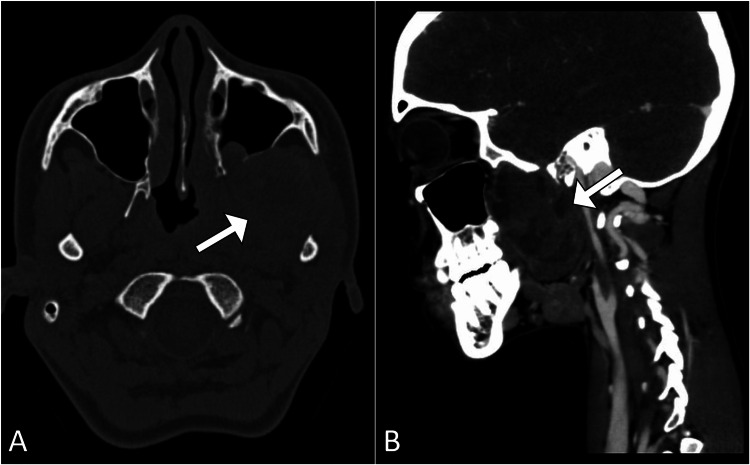
(A) CT scan of the brain demonstrates a soft tissue lesion (arrow) situated in the petrous part of the left temporal bone. (B) After administering contrast, the lesion demonstrates heterogeneous enhancement. CT: computed tomography

**Figure 2:**
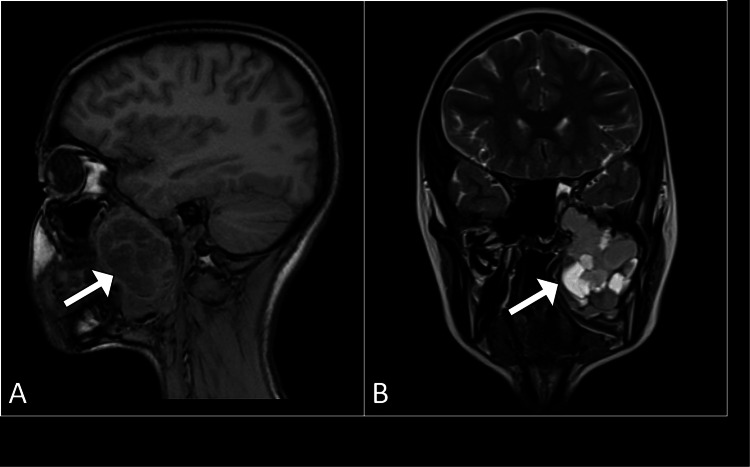
MRI of the brain in sagittal T1-weighted (A) and coronal T2-weighted (B) images, illustrating an expansile lesion (arrows) in the petrous part of the left temporal bone. The mixed signal intensities and fluid-fluid levels suggest the presence of blood products, consistent with an aneurysmal bone cyst. MRI: magnetic resonance imaging

**Figure 3:**
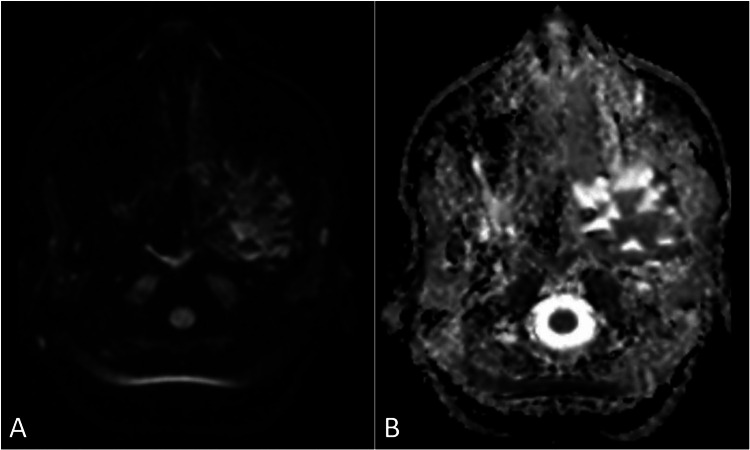
Diffusion-weighted imaging (A) with apparent diffusion coefficient map (B) demonstrates that there is no restricted diffusion observed in the petrous bone lesion.

**Figure 4:**
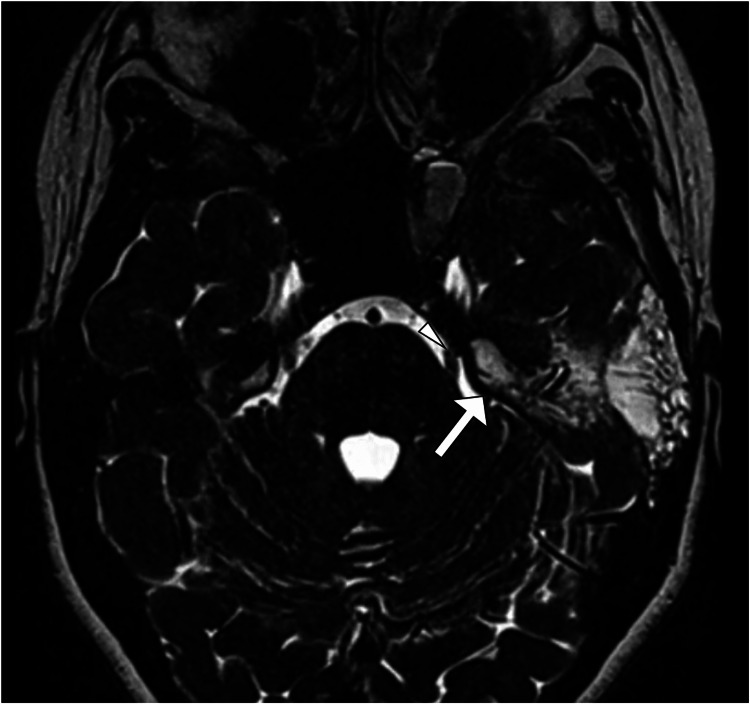
MRI of the brain in the axial plane using the CISS sequence, revealing the lesion of the petrous bone (arrow) in close proximity to the left trigeminal nerve (arrowhead), which can explain the patient's symptoms. MRI, magnetic resonance imaging; CISS, constructive interference in steady state

A request was made for a computed tomography scan of the head, which showed a soft tissue lesion within the petrous part of the left temporal bone that exhibited heterogeneous contrast enhancement (Figure 1). Subsequently, magnetic resonance imaging of the brain was performed using contrast to better characterize the lesion. It revealed an expansive, lobulated mass situated in the petrous region of the left temporal bone. The mass exhibited cysts containing fluid with levels suggestive of blood products. The lesion demonstrated heterogeneous enhancement with hemorrhagic regions, and the cystic spaces showed minimal peripheral enhancement (Figure 2). Its dimensions were approximately 2.8 cm × 2.4 cm × 2.2 cm. The lesion was in close proximity to the trigeminal nerve at the root entry zone (Figure 3). These imaging findings are consistent with a diagnosis of an aneurysmal bone cyst affecting the petrous part of the left temporal bone (Figure 4).

The patient was referred to a tertiary center for further evaluation and management. Given the patient's refractory symptoms and the imaging findings, a decision was made to perform a left retrosigmoid craniotomy with the resection of the lesion. Intraoperative findings revealed a cystic lesion with a thick fibrous wall that was intimately associated with the petrous part of the left temporal bone and the trigeminal nerve. The lesion was resected en bloc, and histopathology confirmed the diagnosis of an aneurysmal bone cyst.

Postoperatively, the patient experienced significant improvement in her facial pain, with the resolution of her numbness and tingling sensations. At the six-month follow-up, the patient reported no recurrence of symptoms. She was able to resume her daily activities and reported a significant improvement in her quality of life. She was advised to continue follow-up appointments to monitor for any recurrence or complications.

## Discussion

Trigeminal neuralgia is a severe condition that causes facial pain and can significantly impact a patient's quality of life [1]. While most cases are idiopathic, some structural causes, including vascular compression, tumors, and cysts, have been reported [1]. In this case report, we present a rare case of trigeminal neuralgia caused by an aneurysmal bone cyst involving the petrous part of the left temporal bone.

Aneurysmal bone cysts are benign bone tumors that typically occur in children and adolescents, primarily in the long bones [2]. They are uncommon in the craniofacial region, accounting for less than 1% of all craniofacial bone tumors. The pathogenesis of aneurysmal bone cysts is not well understood, but they are thought to arise from a combination of osteoclastic and hemorrhagic events [2]. Clinically, aneurysmal bone cysts can present with pain, swelling, and bony deformity and can be challenging to diagnose due to their nonspecific symptoms [2].

The association between trigeminal neuralgia and petrous bone lesions is exceedingly rare, with only a few cases reported in the literature [3,4]. In such cases, the lesions involved the petrous part of the temporal bone and were found to be closely associated with the trigeminal nerve. The mechanism by which aneurysmal bone cysts cause trigeminal neuralgia is unclear, but it is thought to be related to the compression, irritation, or distortion of the trigeminal nerve by the expanding lesion [5].

In our case, the patient had a long-standing history of trigeminal neuralgia refractory to medical management. Imaging studies revealed an aneurysmal bone cyst involving the petrous part of the left temporal bone, causing bony destruction and compression of the trigeminal nerve at the root entry zone. The patient underwent a left retrosigmoid craniotomy with the resection of the lesion, resulting in significant improvement in her facial pain and sensory deficits.

The treatment of aneurysmal bone cysts involving the skull base is challenging due to their location and proximity to critical neurovascular structures [6,7]. Surgical resection is often the treatment of choice, but it may be associated with significant morbidity [2,6]. In addition, the risk of recurrence is high. Other treatment modalities, including embolization, radiation therapy, and sclerotherapy, have been reported, but their efficacy and safety remain unclear [1,2,6].

## Conclusions

Trigeminal neuralgia caused by an aneurysmal bone cyst involving the petrous part of the left temporal bone is a rare but debilitating condition. Diagnosis can be challenging, and a multidisciplinary approach is necessary for effective management. Surgery is the most effective treatment option, and the complete resection of the cyst with the preservation of the surrounding structures should be the goal. Close follow-up is necessary due to the potential for recurrence, but long-term outcomes are generally favorable. With increased awareness and appropriate management, patients with this rare condition can achieve good outcomes and improve their quality of life.
